# When the Heart Pays for the Chest: A Case Report of an Extracardiac Mediastinal Mass Presenting With Cardiac Arrhythmias

**DOI:** 10.7759/cureus.115023

**Published:** 2026-08-23

**Authors:** Loubna El Khadir, Soukaina Chaari, Emane Mengue Phalex, Sara Ahchouch, Aatif Benyass

**Affiliations:** 1 Cardiology, Ibn Sina University Hospital, Rabat, MAR; 2 Cardiology, Military Hospital Mohamed V, Rabat, MAR

**Keywords:** arrhythmias, extra-cardiac mediastinal tumor, left atrium compression, primary mediastinal hepatoid adenocarcinoma, thoracic imaging

## Abstract

Mediastinal masses are an uncommon finding in the emergency department and cause a variety of symptoms and presentations. Cardiac arrhythmias represent a rare complication that may reveal these tumors, particularly through cardiac compression. We report the case of a 40-year-old patient with a past medical history of a primary mediastinal hepatoid adenocarcinoma treated with chemotherapy. The patient was admitted to the emergency department with palpitations occurring with changes of position. Upon further investigation and initial treatment with an oral beta-blocker, the patient had an enormous anterior and middle mediastinal mass extending vertically across all three anatomical levels, with significant mass effect on cardiac structures explaining his dysrhythmia occurring at the change of position. Given the size of the mass and its close relationship to surrounding structures, the patient was started on chemotherapy while continuing beta-blocker therapy. Palpitations are most often a symptom of a benign underlying condition; however, they can also be a sign of potentially serious and life-threatening diseases, and hence a thorough investigation is important.

## Introduction

Palpitations are one of the most common symptoms among patients visiting the emergency department (ED), with causes ranging from benign to life-threatening [1]. They are characterized by the sensation of irregular, rapid, or forceful heartbeats in the chest or a pounding sensation in the neck [2].

Extracardiac mediastinal masses, although rare, may present with palpitations and cardiac dysrhythmias through various pathophysiological mechanisms. Beyond direct myocardial irritation or infiltration, these masses may induce arrhythmias through mechanical compression of cardiac structures, particularly the atria, resulting in local myocardial stretch and alterations in electrophysiological properties through mechano-electrical feedback. Compression of adjacent neural pathways or coronary vessels may also contribute to cardiac dysrhythmias. Depending on the histological type, size, and anatomical relationships of the mass, management may require surgical excision, chemotherapy, or radiotherapy, with treatment directed at relieving the underlying mechanical or pathological cause [3].

## Case presentation

A 40-year-old man was admitted to our intensive care unit for palpitations occuring at change of position. The episodes were triggered when assuming the sitting or lateral decubitus positions, and relieved when moving into the supine position. He had a past medical history of a primary mediastinal hepatoid adenocarcinoma, initially revealed by an episode of hemoptysis with pleural effusion, and confirmed by histopathological examination. He received a total of three cycles of chemotherapy. Seven months after completing his last chemotherapy cycle, he presented with position-dependent palpitations, leading to his current admission.

**Figure 1:**
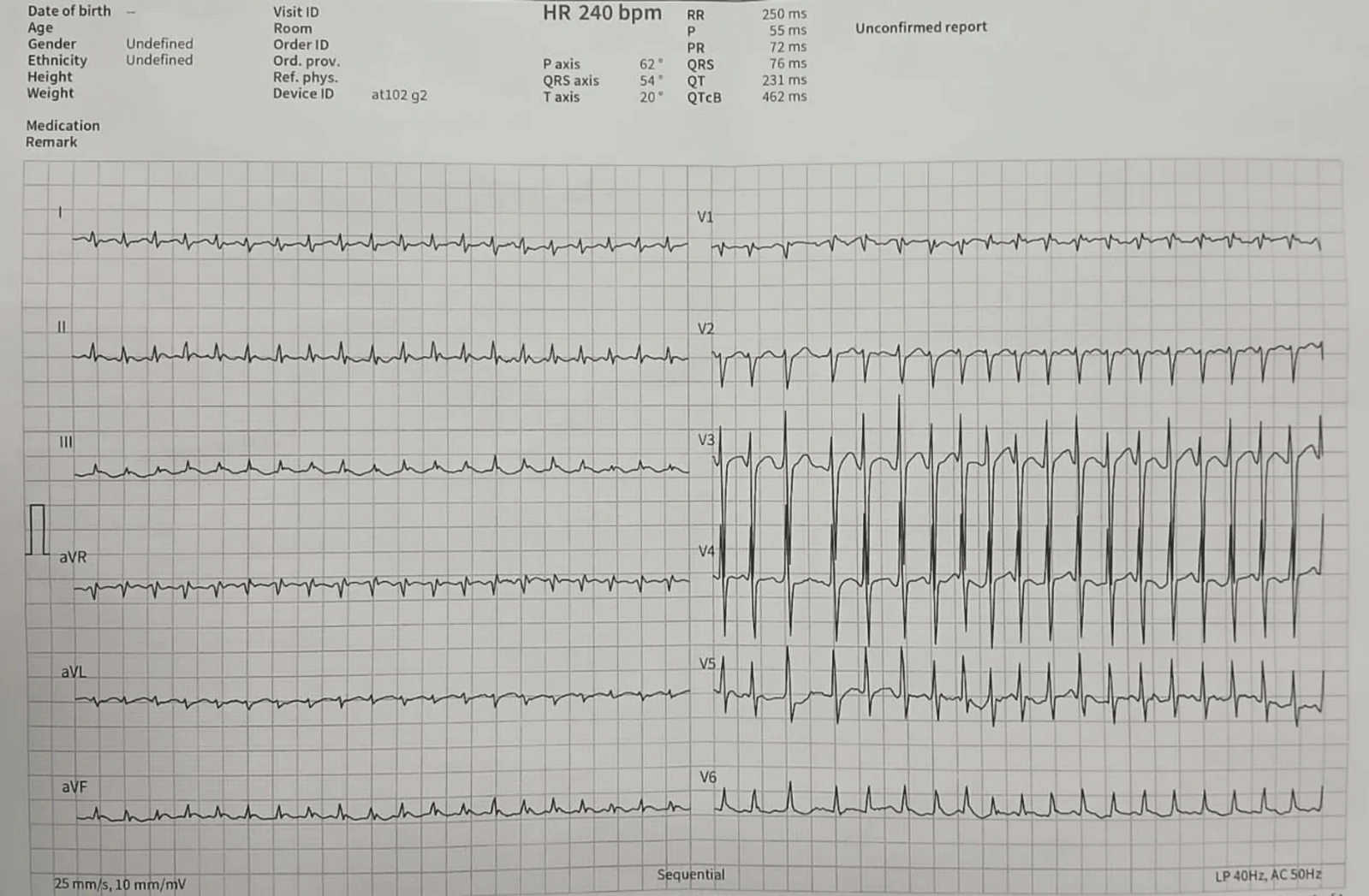
Resting electrocardiogram showing a regular narrow-complex sinus tachycardia with a heart rate reaching 240 bpm. P waves are discernible before each QRS complex, with a 1:1 atrioventricular relationship and a normal P-wave axis (+62°). The PR interval is 72 ms, and the QRS complexes are narrow (76 ms).

**Figure 2:**
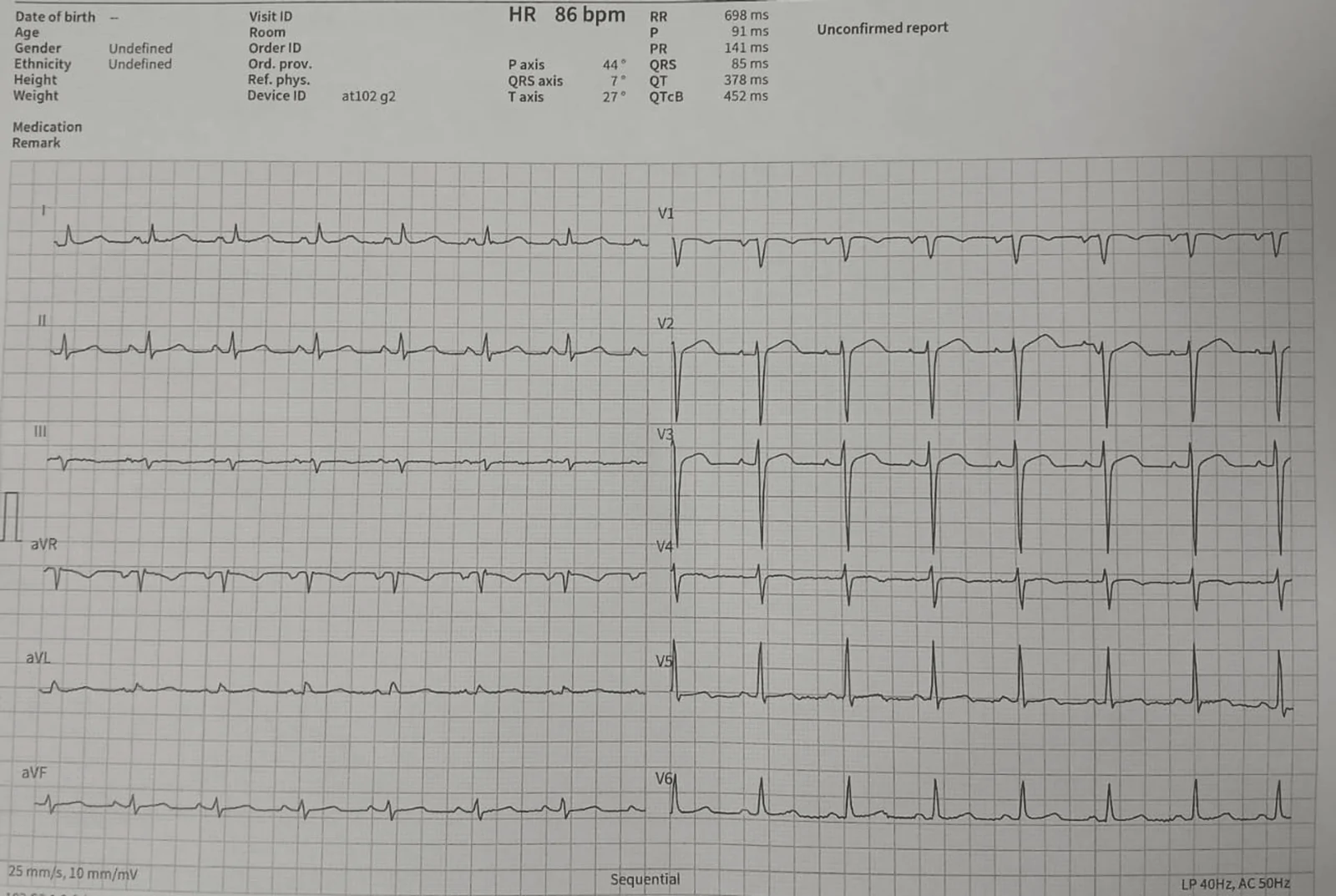
Resting electrocardiogram performed after changing the patient’s position

Clinical examination revealed a conscious, hemodynamically stable patient with a heart rate (HR) of 240 beats per minute (bpm) and a blood pressure (BP) of 110/70 mmHg. By changing to a supine position, the patient's heart rate slowed down, with a heart rate reaching 86 bpm (Figures 1, 2).

This position-dependent phenomenon was consistently reproducible during serial bedside clinical evaluations. Continuous electrocardiographic monitoring demonstrated abrupt onset of tachycardia following a change in position from supine to sitting or lateral decubitus, followed by rapid and spontaneous termination upon returning to the initial position.

Cardiovascular examination showed no signs of left or right heart failure and a normal cardiac auscultation. Tumor marker assays showed an increase in alpha-fetoprotein to more than 20,000 ng/mL. A dose of 5 mg of bisoprolol was initiated once daily upon admission.

**Figure 3:**
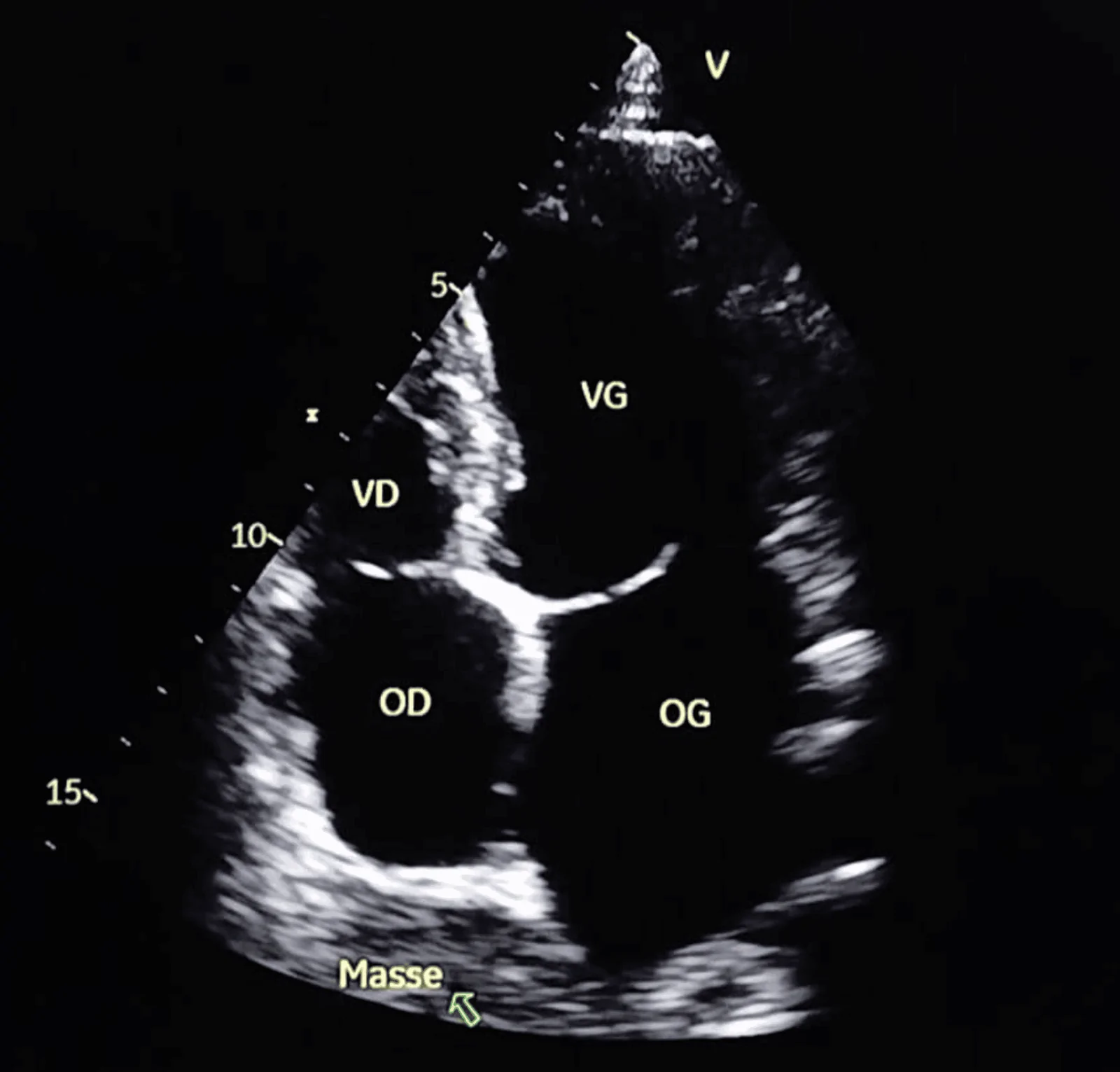
Apical four-chamber echocardiographic view revealing a large mediastinal mass compressing the cardiac structures. OD: *l'oreillette droite* (right atrium); VD: ventricule droit (right ventricle); OG: *oreillette gauche* (left atrium); VG: *ventricule gauche* (left ventricle); Masse: mass (Original labels are in French)

**Figure 4:**
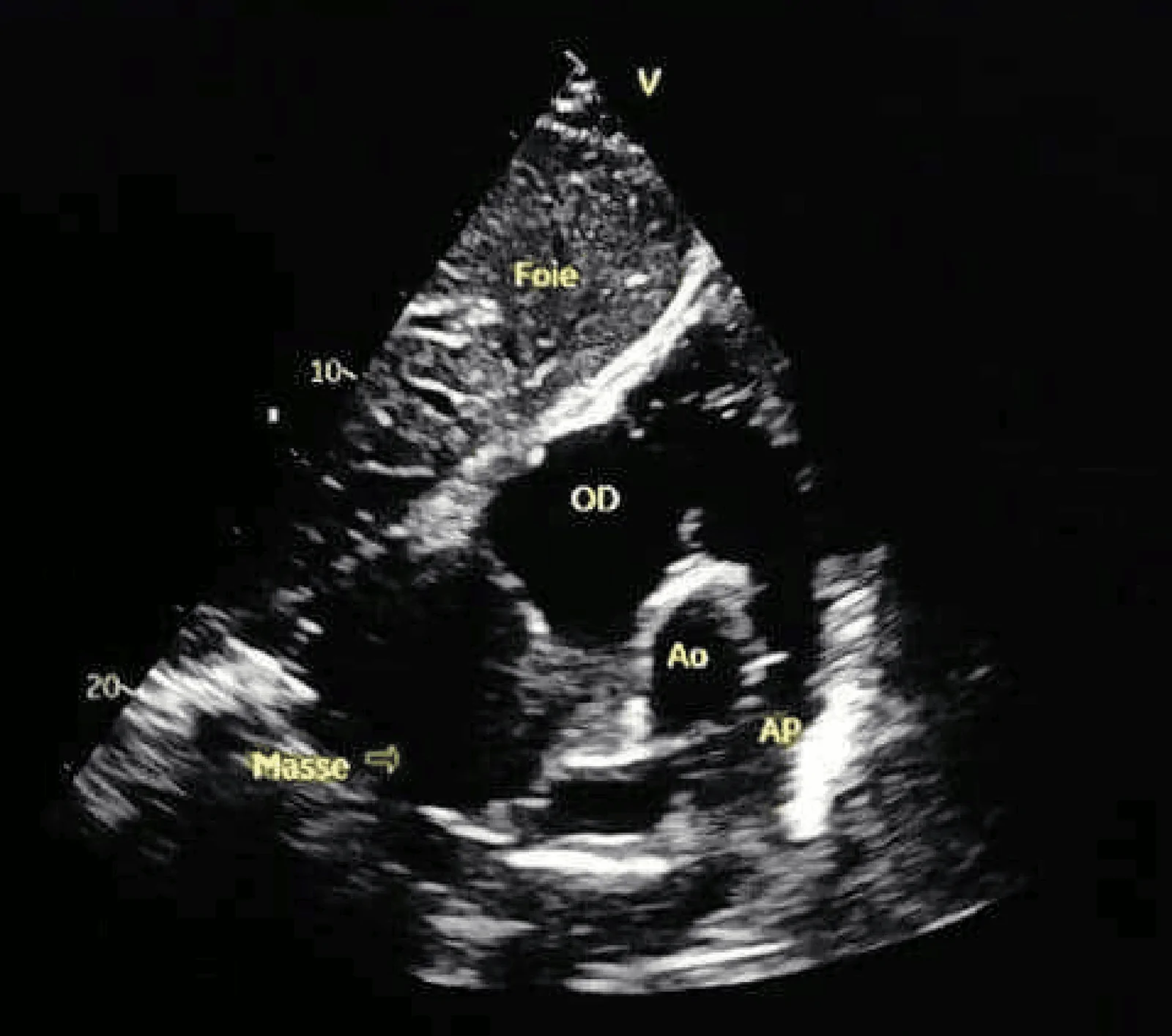
Subcostal echocardiographic view revealing a large mediastinal mass compressing the cardiac structures OD: *l'oreillette droite* (right atrium); OD: *l'oreillette droite* (right atrium); AO: aorta; AP: *artère pulmonaire* (pulmonary artery); *foie*: liver; *Masse*: mass (Original labels are in French)

Transthoracic echocardiography showed a mass compressing the roof of both atria, the right branch of the pulmonary artery, and the superior vena cava, measuring 7 × 7 cm (Figures 3, 4). Left and right ventricular systolic functions were preserved, with a left ventricular ejection fraction (LVEF) of 60% and normal right ventricular function (tricuspid annular plane systolic excursion (TAPSE) 22 mm). Doppler evaluation demonstrated no inflow obstruction across the atrioventricular valves or outflow tract gradient, and the estimated pulmonary artery systolic pressure (PASP) was normal at 11 mmHg with no altered venous flow dynamics.

**Figure 5:**
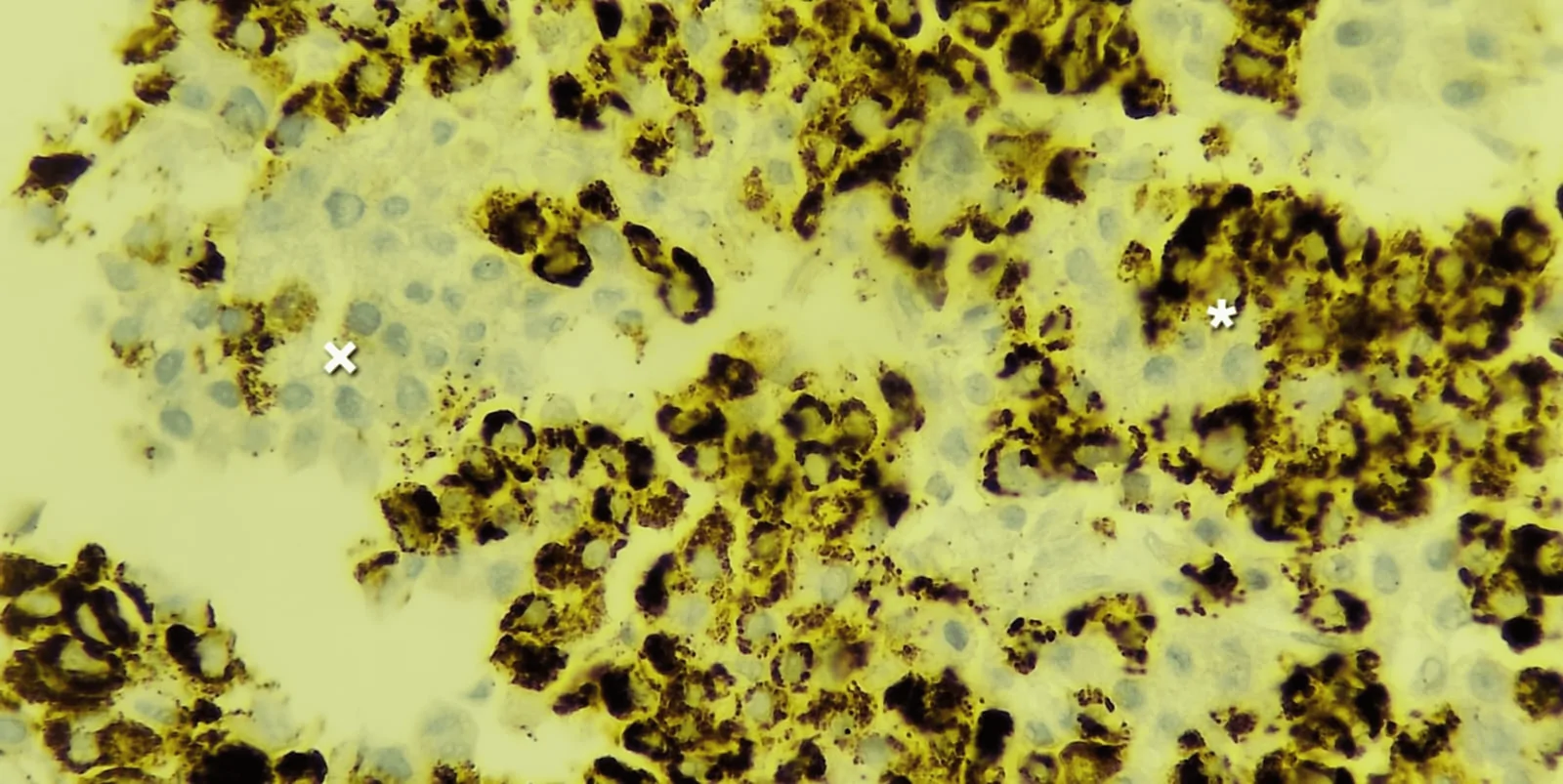
Photomicrograph of hepatoid adenocarcinoma (hematoxylin and eosin strain, ×20) (*) is an area of diverse hepatoid cells, showing a trabecular and solid growth pattern (×) composed of large polygonal cells with abundant eosinophilic and clear cytoplasm

**Figure 6:**
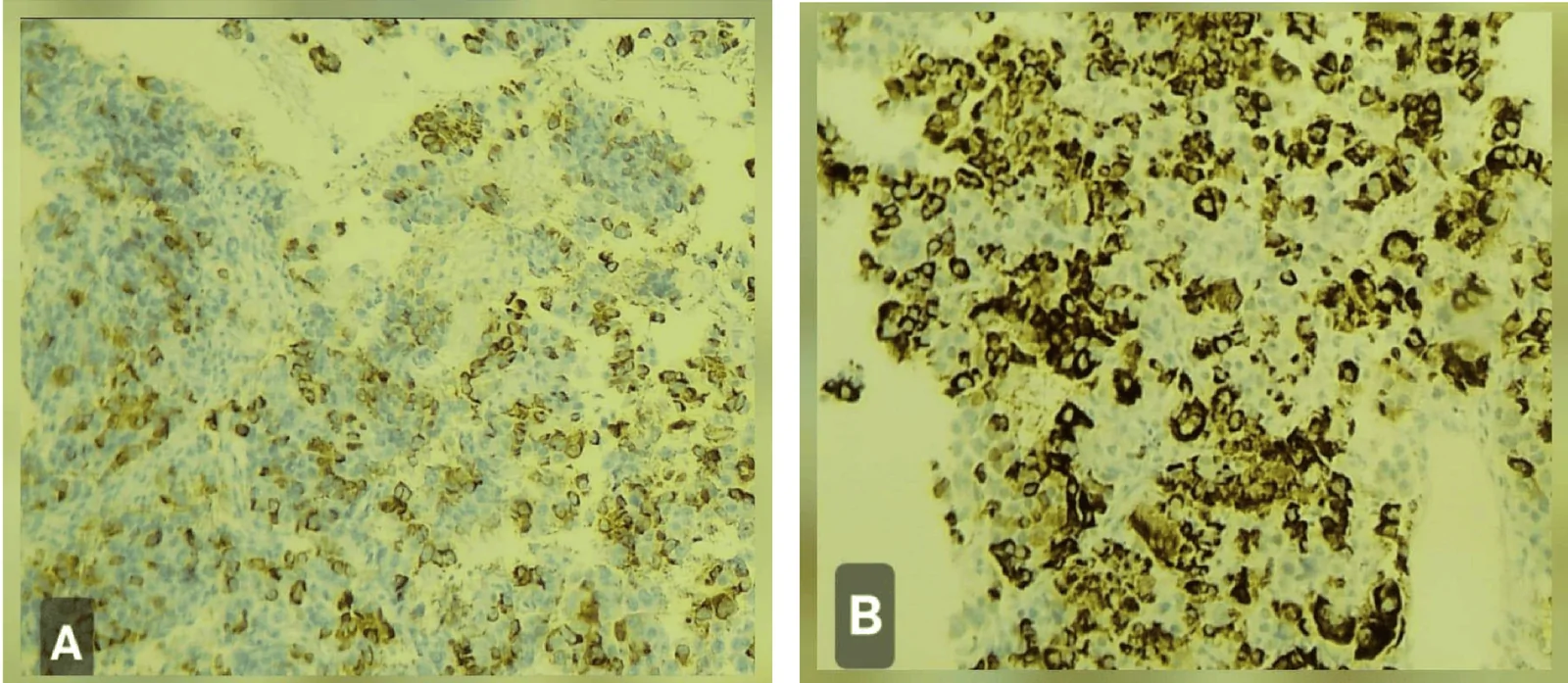
Immunohistochemical staining of hepatoid adenocarcinoma (A) Positive staining for alpha fetoprotein. (B) Positive staining for hepatocyte paraffin 1

The histopathological examination of the performed biopsy was consistent with hepatoid adenocarcinoma (Figures 5, 6).

**Figure 7:**
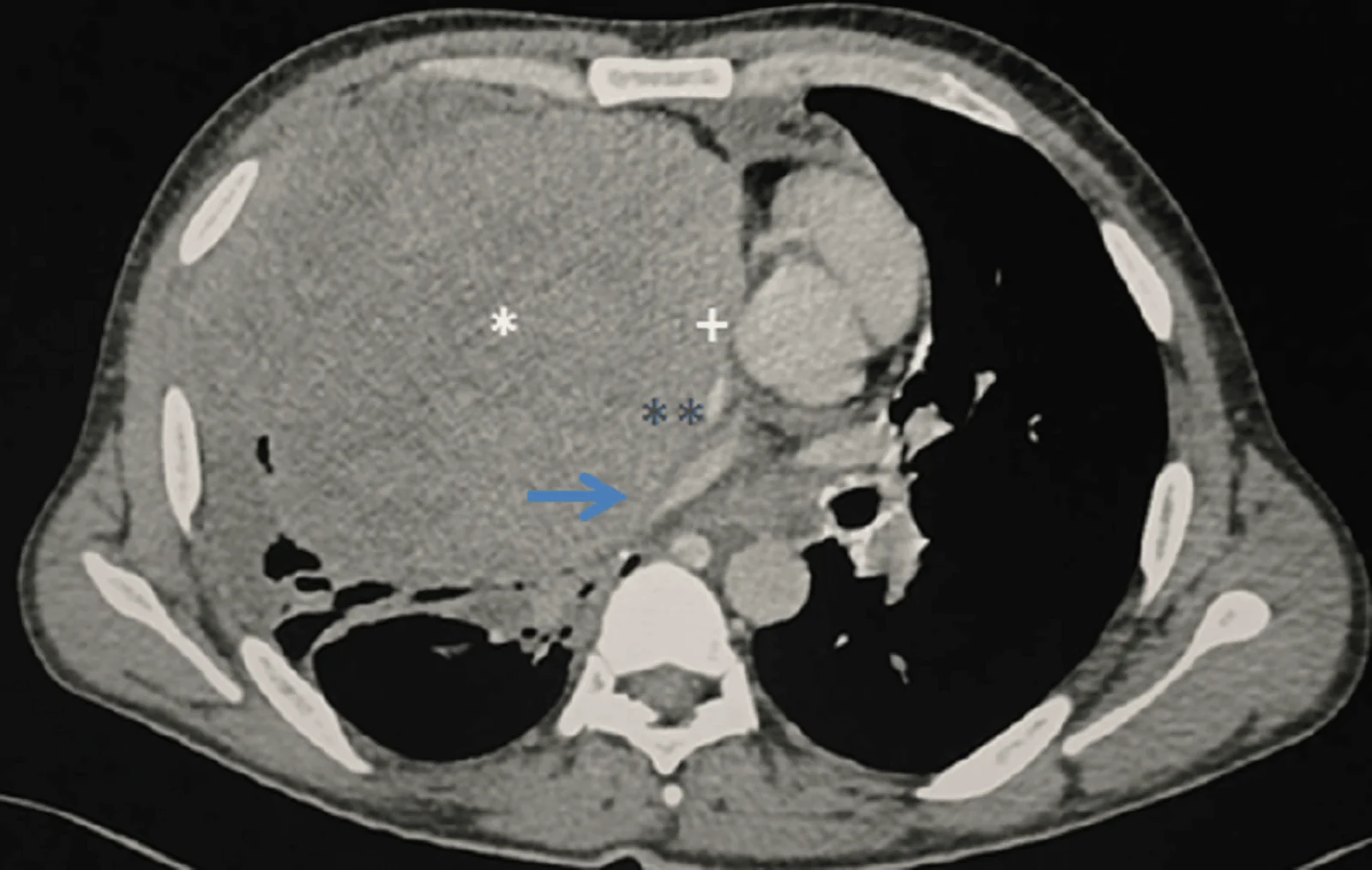
Axial contrast-enhanced chest computed tomography (CT) scan showing a large mediastinal mass (*) with its mass effect in the cardiac structures: The superior vena cava (white plus sign, +), the right main pulmonary artery (blue double asterisk,**), and the right atrium (blue arrow, →)

In parallel, a contrast-enhanced chest computed tomography (CT) scan was indicated to evaluate the anatomical relationships of this large mass. It revealed a voluminous anterior and middle mediastinal mass measuring 13 × 15 × 19.5 cm, spanning vertically across all three anatomical levels (superior, middle, and inferior mediastinum). The mass exerted significant compression on adjacent cardiovascular structures, notably the superior vena cava (SVC), the right atrium (RA), the right pulmonary veins, and the right main pulmonary artery (Figure 7). The CT scan demonstrated a completely normal hepatic parenchyma with no evidence of focal liver lesions, masses, or chronic liver disease, supporting the diagnosis of a primary mediastinal hepatoid adenocarcinoma.

**Figure 8:**
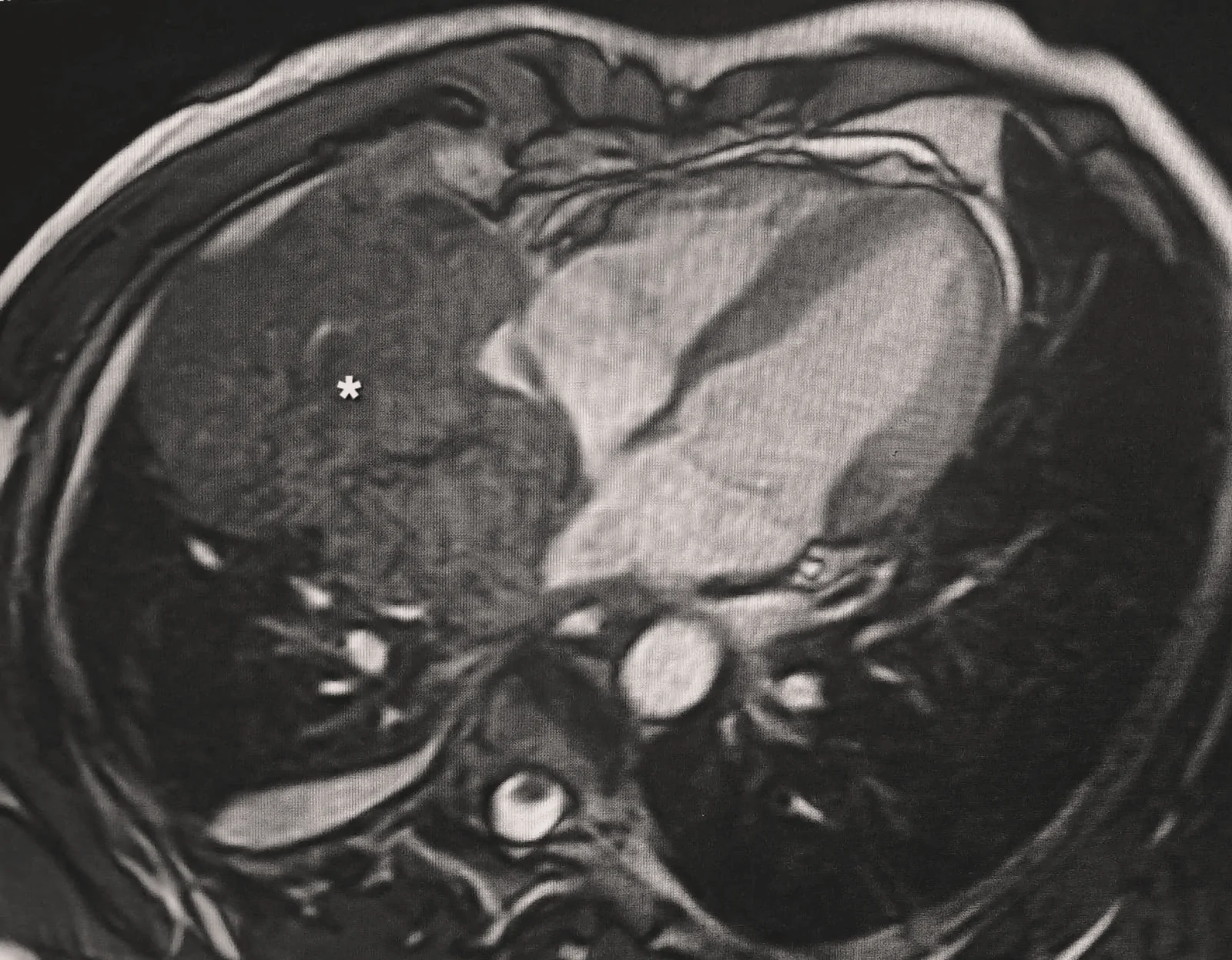
Four-chamber cine bSSFP CMR image showing anatomical relationships of the mediastinal tumor (*) with cardiac structures CMR: cardiac magnetic resonance; bSSFP: balanced steady-state free precession

Cardiac magnetic resonance imaging (CMR) confirmed the presence of the enormous extracardiac mediastinal mass compressing the RA, the SVC, the right pulmonary veins, and the right main pulmonary artery, causing a significant mass effect and leftward cardiac shift. The underlying atrial and ventricular myocardium showed preserved borders without direct myocardial invasion (Figure 8).

Due to the close relationship of the mediastinal mass and its enormous size, the therapeutic decision was to complete chemotherapy sessions and continue treatment with beta-blockers. A dose of 5 mg of bisoprolol was maintained, achieving effective heart rate control (resting sinus rate: 60-75 bpm) and complete resolution of positional tachycardia. The treatment was well tolerated without adverse effects, and hemodynamic stability was maintained over an eight-month follow-up.

## Discussion

Arrhythmias directly induced by non-cardiac primary tumors are rare [4]. Mediastinal tumors can affect cardiac electrical activity either directly or by causing systemic abnormalities, such as autonomic nervous system disorders and inflammation [5]. We presented the case of a young patient with position-dependent arrhythmia, where clinical and radiological findings strongly suggested a potential mechanical relationship secondary to mass effect from a giant mediastinal hepatoid adenocarcinoma.

Primary anterior mediastinal masses most commonly comprise thymomas, teratomas, ectopic thyroid tumors, and lymphomas, making primary mediastinal hepatoid adenocarcinoma an extraordinarily rare entity. The characterization of a mediastinal mass requires a stepwise approach, beginning with conventional radiography, contrast-enhanced CT or magnetic resonance imaging (MRI), and biopsy for histopathological evaluation, which remains essential for accurate diagnosis and treatment planning [6].

Transthoracic echocardiography remains the imaging modality of choice for evaluating the functional and hemodynamic impact of mediastinal masses. It provides a detailed assessment of volumetric changes, segmental wall motion abnormalities, and intracavitary pressure gradients. While anterior mediastinal masses preferentially compress the right cardiac chambers, posterior masses predominantly affect the left atrium. When arrhythmias occur, such localized cardiac compression underscores the role of mechano-electrical feedback in triggering these events [7,8].

Panda et al. demonstrated that MRI, particularly cine imaging, allows a more precise assessment of cardiovascular involvement by mediastinal masses when CT findings are equivocal, accurately identifying invasion or adherence to cardiac structures with high sensitivity and specificity [9].

Parambil et al. reported a case of left atrial compression by a large posterior mediastinal mass, a condition with a well-recognized potential for catastrophic hemodynamic compromise. Notably, the initial clinical presentation was a sudden onset of paroxysmal atrial fibrillation, which was likely related to mechanical stretching of the atrial myocardial fibers induced by the extrinsic compression [10]. Dúbrava et al. reported the case of a mediastinal lymphoma that caused compression of the left atrium and superior vena cava [11].

A notable aspect of this case was the position-dependent sinus tachycardia, an uncommon presentation that is rarely described in the literature, which responded favorably to beta-blocker therapy. In our patient, transthoracic echocardiography demonstrated positional cardiac compression temporally associated with the onset of tachycardia, providing a potential mechanistic link between the extracardiac mass and the arrhythmia. This finding highlights the value of echocardiography in assessing dynamic cardiac compression, chamber distortion, and associated hemodynamic consequences.

## Conclusions

Cardiac arrhythmias secondary to mediastinal masses are rare but may manifest as position-dependent sinus tachycardia. Beta-blockers may provide effective symptomatic and rate control as a bridge to mass-targeted therapy. Close clinical and rhythm monitoring is essential to assess the resolution of arrhythmias following treatment.
